# Drug–drug interactions between letermovir and tacrolimus in Japanese renal transplant recipients simulated using a physiologically based pharmacokinetic model

**DOI:** 10.3389/fmicb.2024.1480874

**Published:** 2024-10-08

**Authors:** Takumi Maruyama, Hidefumi Kasai, Yutaka Fukaya, Mitsuru Shiokawa, Toshimi Kimura, Yukihiro Hamada

**Affiliations:** ^1^Department of Pharmacy, Tokyo Women's Medical University Hospital, Tokyo, Japan; ^2^Department of Pharmacy, Kochi Medical School Hospital, Kochi, Japan; ^3^Keio Frontier Research and Education Collaboration Square (K-FRECS) at Tonomachi, Keio University, Kanagawa, Japan; ^4^Department of Pharmacy, Juntendo University Hospital, Tokyo, Japan

**Keywords:** physiologically based pharmacokinetic modeling, drug–drug interaction, letermovir, tacrolimus, renal transplantation, Japanese population

## Abstract

Letermovir (LET) is a novel antiviral agent recently approved for cytomegalovirus (CMV) prophylaxis of renal transplant patients in Japan. However, its interactions with tacrolimus (TAC), an important immunosuppressant, remain ambiguous, warranting careful evaluation considering the unique genetic and physiological characteristics of Japanese patients. Therefore, in this study, we aimed to investigate the drug–drug interactions between LET and extended-release TAC (ER-TAC) in Japanese renal transplant patients via physiologically based pharmacokinetic (PBPK) modeling. We developed PBPK models for LET and TAC, including a new model for ER-TAC, using the Simcyp simulator. We also created a virtual Japanese post-transplant population by incorporating physiological parameters specific to Japanese patients, including CYP3A5 genotypes. Our model accurately predicted the pharmacokinetics of both immediate-release and ER-TAC co-administered with LET. In the Japanese population, LET significantly increased ER-TAC exposure, with the effect varying by CYP3A5 genotype. For CYP3A5*1 carrier, the area under the curve ratio ranged from 2.33 to 2.53, while for CYP3A5*3/*3 carriers, it ranged from 2.82 to 2.86. The maximum concentration ratio was approximately 1.50 across all groups. Our findings suggest reducing the ER-TAC dose by approximately 57–60% for CYP3A5*1 carrier and 65% for CYP3A5*3/*3 carriers when co-administered with LET for Japanese renal transplant patients. Moreover, the developed model incorporating population-specific factors, such as hematocrit values and CYP3A5 genotype frequencies, is a valuable tool to evaluate complex drug interactions and guide the dosing strategies for LET and TAC in Japanese patients. Overall, this study expands the application of PBPK modeling in transplant pharmacology, contributing to the development of effective immunosuppressive strategies for Japanese renal transplant patients.

## 1 Introduction

Application of letermovir (LET), a novel antiviral agent initially developed for cytomegalovirus (CMV) prophylaxis of allogeneic hematopoietic stem cell transplant patients, has recently been expanded for CMV prophylaxis of renal transplant patients in Japan (Ishida et al., 2024). LET undergoes complex metabolism, mainly via glucuronidation by uridine 5′-diphospho-glucuronosyltransferase (UGT)-1A1/3, along with oxidative metabolism by cytochrome P450 (CYP)-3A4 (Menzel et al., 2021). It exhibits both inhibition and weak induction of CYP3A4 and inhibition of UGT1A1 (McCrea et al., 2019). Tacrolimus (TAC), an important immunosuppressant, is mainly metabolized by CYP3A4 and CYP3A5, along with glucuronidation by UGT1A4, and inhibits UGT1A1 (Laverdière et al., 2011; Sun et al., 2012). The metabolic profiles of LET and TAC, particularly LET’s effects on CYP3A4 and TAC’s metabolism by both CYP3A4 and CYP3A5, suggest potential drug–drug interactions (DDIs) when used in combination (Emoto et al., 2019). However, their combined effects, particularly in Japanese renal transplant patients (Satoh et al., 2008), remain unclear, warranting further investigation.

Although some studies have investigated the DDIs between LET and TAC (McCrea et al., 2019), comprehensive investigations in Japanese renal transplant patients are lacking. Moreover, only a few studies have assessed the interactions between extended-release (ER)-TAC and LET (Limaye et al., 2023). Effects of Japanese population-specific genetic polymorphisms, particularly of the CYP3A5*1 allele, on these drug interactions also remain unclear. Additionally, effect to LET metabolism, UGT1A1 inhibition by TAC requires further investigation. These knowledge gaps cause difficulties in dose adjustment of LET and TAC and treatment optimization of Japanese renal transplant patients. Comprehensive evaluation of the pharmacokinetic interactions of drugs is necessary considering the characteristics specific to Japanese patients. Therefore, in this study, we aimed to quantify the effects of LET on ER-TAC pharmacokinetics in Japanese renal transplant patients via physiologically based pharmacokinetic (PBPK) modeling.

## 2 Materials and methods

### 2.1 PBPK model development

PBPK models were developed using Simcyp simulator version 23 (Certara Inc., Sheffield, United Kingdom). For LET, we used a predefined Simcyp model that accounted for both CYP3A4 inhibition and induction. We extended this model by including the reported inhibition constant (Ki) of UGT1A1 (Menzel et al., 2023).

For TAC, we developed models for both immediate-release (IR) and ER formulations. These models were developed using previously reported *in vitro* and *in vivo* data (Emoto et al., 2019), including absorption rates, distribution volumes, clearance (CL) values, and enzyme kinetics of CYP3A4 and CYP3A5. For the ER-TAC model, we incorporated additional *in vitro* dissolution and permeability data (Sun et al., 2012; Mercuri et al., 2016; Loer et al., 2023).

### 2.2 Virtual population generation

We created a virtual Japanese post-renal transplant population by modifying the pre-existing healthy Japanese adult population data in Simcyp. We included physiological parameters specific to early renal transplant patients, such as hematocrit values, renal function indicators, and serum albumin levels. These parameters were derived from previously published data (Satoh et al., 2008) on Japanese renal transplant patients. We adjusted the abundance of CYP3A as previously described (Itohara et al., 2022).

To incorporate the effects of CYP3A5 phenotype in our PBPK model, we defined individuals carrying the CYP3A5*1 allele as expressers (extensive metabolizers, EM) and those with the CYP3A5*3/*3 genotype as non-expressers (poor metabolizers, PM) in Simcyp. CYP3A5 abundance values for expressers in various tissues (liver, small intestine, and colon) were set based on literature values (Itohara et al., 2022) or Simcyp default values when no specific abundance values were specified. For non-expressers (PM), the CYP3A5 abundance was set to 0. For simulations focusing on specific CYP3A5 phenotypes, we adjusted the frequency of CYP3A5 genotypes in the virtual population accordingly. In cases where no specific genotype distribution was required, we utilized the default CYP3A5 genotype frequencies provided in Simcyp’s built-in population models. Using these settings, we conducted analyses considering CYP3A5 genetic polymorphisms and phenotypes for each simulation scenario.

### 2.3 DDI simulations

We performed two types of DDI simulations following established PBPK modeling practices for DDI prediction in Table 1:

- Comparison of the pharmacokinetics of IR-TAC and ER-TAC co-administered with LET: We simulated ER-TAC pharmacokinetics under the same conditions as those used for the validated model of IR-TAC with LET. We compared their pharmacokinetic parameters, taking into account CYP3A5 genetic polymorphisms, to determine the formulation-specific differences in their interaction with LET.
- Evaluation of the pharmacokinetics of ER-TAC with and without LET in Japanese early renal transplant patients: We created two distinct virtual Japanese post-renal transplant populations based on CYP3A5 genotypes: one with only CYP3A5*1 carrier and another with only CYP3A5*3/*3 carriers. Using these populations, we simulated ER-TAC pharmacokinetics with and without LET for each genotype group to predict the extent of DDIs in these patient populations.

**Table 1:** Simulation conditions for drug–drug interaction studies.

DDI simulation	Route	Population	Age range (year)	Proportion of females	Population size	Dose	Prandial state
IR vs. ER-TAC with LET	po	Healthy volunteers in Simcyp	26–54	1	140 simulations (n = 14 subjects; n = 10 trials)	Multiple oral QD doses of LET 480 mg administered for 16 d with a single oral dose of TAC 5 mg on day 8	Fasted
ER-TAC with and without LET in Japanese early RTx patients	po	Japanese early RTx patients (separate simulations for CYP3A5*1 carrier only and CYP3A5*3/*3 carriers only	26–54	0.5	140 simulations for each genotype group (n = 14 subjects; n = 10 trials)	Multiple oral QD doses of LET 480 mg administered for 16 d with a single oral dose of TAC 5 mg on day 8	Fasted

DDIs, drug–drug interactions; IR-TAC, immediate-release tacrolimus; ER-TAC, extended-release tacrolimus; LET, letermovir; RTx, renal transplant; AUC_inf_, area under the blood concentration–time curve from 0 to infinity; C_max_, maximum concentration; QD, once daily.

In both simulations, we analyzed the key pharmacokinetic parameters, including the area under the curve (AUC), maximum concentration (C_max_), and CL values. Furthermore, we calculated the ratios of these parameters between the LET and TAC combination and TAC alone groups to quantify the extent of their interactions.

### 2.4 Model validation and data analysis

Next, the established models were validated by comparing the simulated pharmacokinetic parameters with published clinical data (Table 2) (Mancinelli et al., 2001; PMDA, 2008; McCrea et al., 2019). Observational data for model validation were extracted from the literature using the GetData Graph Digitizer 2.26 (Wojtyniak et al., 2020). We evaluated the model performance using the ratio of predicted to observed values. We used the following criteria adapted from previous studies: 0.8–1.25 = good predictions, 0.67–1.5 = reasonable predictions, and 0.5–2 = acceptable predictions (Guest et al., 2011; Emoto et al., 2019).

**Table 2:** Simulation conditions for model validation.

No.	Model	Route	Population	Age range (year)	Proportion of females	Population size	Dose	Prandial state	References
1	IR-TAC	iv	Healthy volunteers in Simcyp	25.5–67.3	0.417	120 simulations (n = 12 subjects; n = 10 trials)	Infused 0.015 mg/kg for 4 h	N.D.	Mancinelli et al.
2		po	Healthy volunteers in Simcyp	25.5–67.3	0.417	120 simulations (n = 12 subjects; n = 10 trials)	5 mg, single dose	Fasted	Mancinelli et al.
3	ER-TAC	po	Japanese patients in Simcyp	20–39	0	200 simulations (n = 20 subjects; n = 10 trials)	3 mg/kg, single dose	Fasted	2.7.6.6 Phase I single-dose study (3 mg) [FJ-506E-0001] [In Japanese], PMDA
4	IR-TAC with LET	po	Healthy volunteers in Simcyp	26–54	1	140 simulations (n = 14 subjects; n = 10 trials)	Multiple oral QD doses of LET 480 mg administered for 16 d with a single oral dose of TAC 5 mg on day 8.	Fasted	McCrea et al.

IR-TAC, immediate-release tacrolimus; ER-TAC, extended-release tacrolimus; LET, letermovir; QD, once daily; iv, Intravenous; po, orally administered.

References: Mancinelli et al. (2001), McCrea et al. (2019), and PMDA (2008).

Next, we assessed whether the observed values were within the 5th to 95th percentile range of the simulated data generated by Simcyp. This range represents the variability in the simulated population and provides a more comprehensive view of model performance than point estimate alone.

We also performed visual predictive checks by comparing the simulated concentration–time profiles with the observed data from clinical studies. This qualitative assessment ensured that the model captured the overall pharmacokinetic behaviors of the drugs throughout the dosing period.

Pharmacokinetic parameters were calculated via non-compartmental analysis using Simcyp. To assess DDIs, we calculated the ratios of AUC, C_max_, and CL values for TAC co-administered with LET vs. TAC alone. Additionally, we assessed the magnitude of drug interactions by comparing the ratios of the IR- and ER-TAC formulations.

These analyses provided insights into the variability of drug behaviors among individuals, facilitating the comprehensive evaluation of model performance and extent of DDIs.

### 2.5 Ethical approval

This study utilized data and materials obtained solely from published literature. As no primary data collection or human subjects were involved, ethical approval was not required.

## 3 Results

### 3.1 PBPK model development and validation

#### 3.1.1 LET model

We used the pre-validated LET compound file from Simcyp v23 as the base model. This model has been extensively validated and is widely accepted in the field. The only modification was the incorporation of the UGT1A1 Ki of 16 μM based on previous data (Menzel et al., 2023). Considering the robustness of the base model and minor modifications, further clinical trial simulations and visual predictive checks were unnecessary. The established model showed the comprehensive pharmacokinetic and metabolic profiles of LET and was suitable for DDI analysis.

#### 3.1.2 IR-TAC model

**Figure 1:**
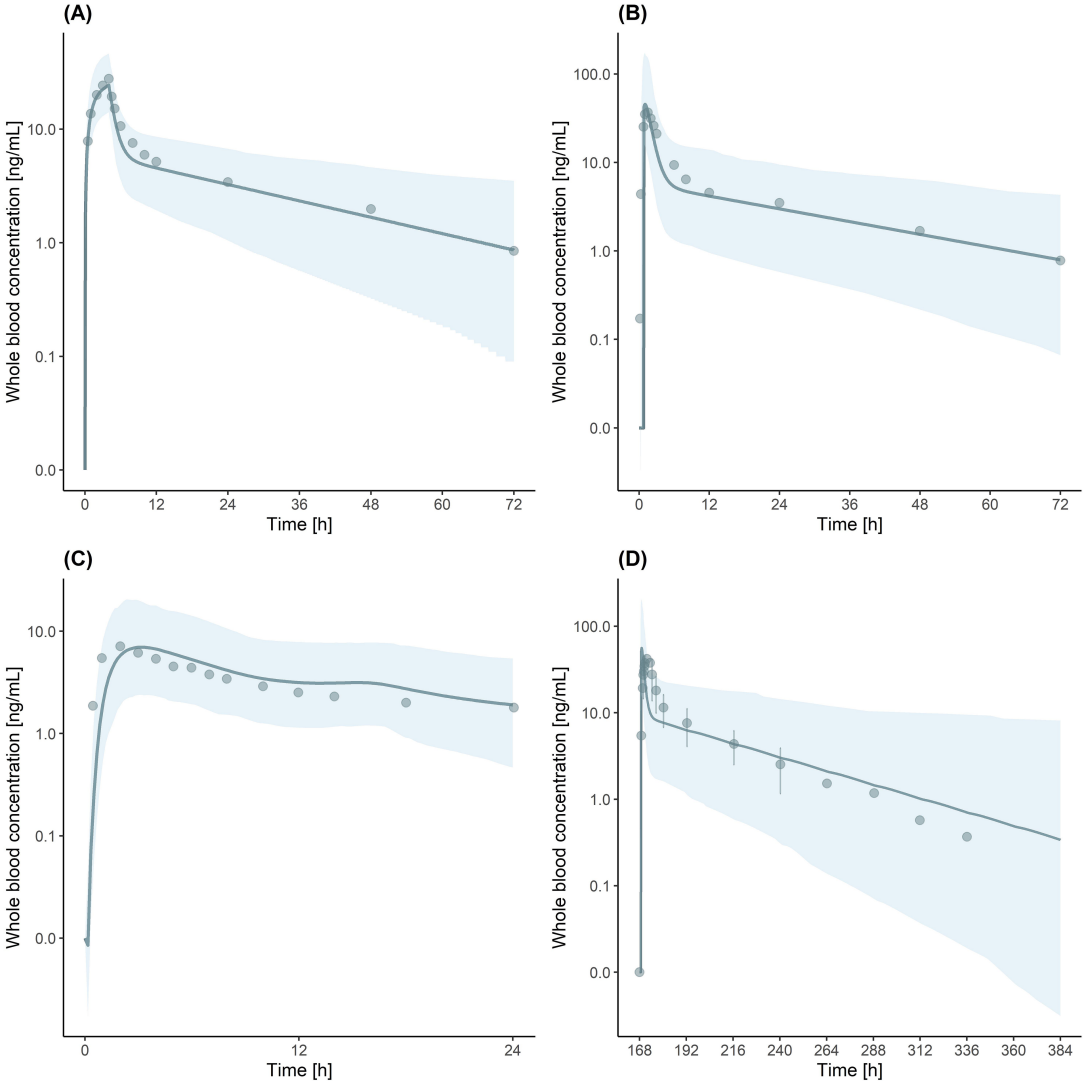
Visual predictive checks for tacrolimus (TAC) physiologically based pharmacokinetic (PBPK) models. **(A)** Immediate-release (IR)-TAC model after intravenous administration. **(B)** IR-TAC model after oral administration. **(C)** Extended-release (ER)-TAC model. **(D)** Drug–drug interaction (DDI) simulation of IR-TAC with letermovir (LET). Solid lines indicate the predicted geometric mean concentration. Shaded regions indicate the 5th to 95th percentile range of simulated data. Observed data points are indicated by dots. Error bars in **(D)** represent the standard deviation in observed data.

We also developed an IR-TAC model using parameters from the literature (Table 3) (Emoto et al., 2019). We incorporated the CYP3A4/5 Ki (Loer et al., 2023) and UGT Ki (Sun et al., 2012) values from recent studies. The established model showed good predictive performance, with ratios of predicted to observed values for the area under the blood concentration–time curve from 0 to infinity (AUC_inf_), C_max_, time to reach C_max_ (T_max_), and CL ranging from 0.77 to 1.25 (Table 4). Visual predictive checks demonstrated good agreement between the predicted and observed concentration–time profiles (Figures 1A,B), further validating the accuracy of the developed model.

**Table 3:** Parameters used for building immediate-release (IR)- and extended-release (ER)-tacrolimus (TAC) physiologically based pharmacokinetic (PBPK) models based on the literature.

Parameter		Value
Physicochemical parameters	Molecular weight (g/mol)	804.0182
	LogP	3.3
	Compound type	Neutral
Blood-binding properties	Fraction unbound in serum	0.012
	Blood-to-plasma ratio	35
	Plasma binding protein	Human serum albumin
Absorption	*First-order model*	
	*f*_a_	1.00
	*k*_a_	3.68
	Lag time (hour)	0.43
	*ADAM*	
	Solubility (pH)	0.01 (7.4)
	Permeability in Caco-2 cells (cm/s)	6.58 × 10^6^
	Dissolved profile	Mean (CV%) values from the *in vivo* dissolved profiles reported in the literature
Distribution	*Minimal PBPK model*	
	*k*_in_ (1/h)	0.68
	*k*_out_ (1/h)	0.1
	*V*_sac_ (L/kg)	10.8
	Predicted *V*_ss_ (L/kg)	17.1
Elimination	*CYP kinetic parameters*	
	CYP3A4 Km (μM)	0.21
	CYP3A4 *V*_max_ (pmol/min/pmol CYP)	3.8
	CYP3A5 Km (μM)	0.21
	CYP3A5 V_max_ (pmol/min/pmol CYP)	2.5
	Renal clearance (mL/min)	0.014
Interaction	CYP3A4 K_i_ (μmol/L)	0.04
	CYP3A4 K_I_ (μmol/L)	2.66
	CYP3A4 K_inact_ (1/min)	0.3
	CYP3A5 K_I_ (μmol/L)	2.69
	CYP3A5 K_inact_ (1/min)	0.21
	UGT1A1 K_i_ (μmol/L)	4.7

IR, immediate-release; ER, extended-release; CYP, cytochrome P450; *f*_a_, fraction available from dosage form; *k*_a_, first-order absorption rate constant; *k*_in_ and *k*_out_, first-order rate constants describing the transfer of tacrolimus to a single adjusting compartment; ADAM, advanced dissolution, absorption, and metabolism; K_m_, Michaelis–Menten constant; PBPK, physiologically based pharmacokinetic; *V*_max_, maximum rate of metabolite formation; *V*_sac_, volume of single adjusting compartment; *V*_ss_, volume of distribution at steady state using tissue volumes of a population of healthy volunteers; K_i_, inhibition kinetic parameter; K_I_, inactivation constant; K_inact_, maximum inactivation rate constant.

For the dissolution profile, the 24-h values reached a plateau. Therefore, the same values as the 16-h measurement values were used.

**Table 4:** Comparison of the predicted and observed pharmacokinetic parameters for model validation.

No.	Model	AUC_inf_ (ng/mL*h)			C_max_ (ng/mL)			T_max_ (h)			CL (L/h/kg or L/h)		
		Pred	Obs	Pred/Obs	Pred	Obs	Pred/Obs	Pred	Obs	Pred/Obs	Pred	Obs	Pred/Obs
1	IR-TAC, iv	339	321	1.06	24.43	26.4	0.93	N.D.	N.D.	N.D.	0.040	0.046	0.87
2	IR-TAC, po	323	293	1.10	47.17	37.8	1.25	1.00	1.30	0.77	0.21	0.25	0.84
3	ER-TAC	174.0	150.8	1.15	7.55	6.8	1.11	3.03	2.60	1.17	N.D.	N.D.	N.D.
4	IR-TAC with LET	908 (749–1,101)	697 (540–899)	1.30 (1.38–1.22)	58.1 (50.8–66.5)	51.5 (44.1–60.0)	1.13 (1.15–1.11)	1.04 (0.75–1.50)	3.00 (1.00–5.00)	0.35 (0.75–0.3)	5.51 (166.0)	7.38 (44.0)	0.74 (2.63)

IR-TAC, immediate-release tacrolimus; ER-TAC, extended-release tacrolimus; LET, letermovir; AUCinf, area under the blood concentration–time curve from 0 to infinity; Cmax, maximum concentration. T_max_, time to maximum concentration; CL, clearance; Pred, predicted value; Obs, observed value.

The “No.” column refers to the respective simulation numbers assigned in Table 2. AUC_inf_, C_max_, and CL values are represented as geometric means with 95% confidence intervals (95% CIs). T_max_ values are represented as medians with ranges.

#### 3.1.3 ER-TAC model

Next, ER-TAC model was developed based on the IR-TAC model, with modifications to the absorption parameters, using published *in vitro* and *in vivo* data on ER formulations (Mercuri et al., 2016; Loer et al., 2023). The established model exhibited good accuracy, with prediction ratios ranging from 1.00 to 1.17 for the area under the blood concentration–time curve from 0 to 24 h (AUC_0-24_), C_max_, and T_max_ values (Table 4). Visual predictive checks showed that the model accurately captured the ER pharmacokinetic profile, with the observed data points falling within the predicted 5^th^ to 95^th^ percentile range (Figure 1C).

#### 3.1.4 DDI simulation of IR-TAC with LET

DDI model was validated using clinical trial data (McCrea et al., 2019). The simulation accurately predicted the interaction between IR-TAC and LET, with predicted/observed ratios of AUC_inf_, C_max_, and CL ranging from 0.74 to 1.30 (Table 4). Visual predictive checks showed that most of the observed concentration–time data points fell within the predicted 5^th^ to 95^th^ percentile range (Figure 1D). However, a slight discrepancy was observed in T_max_, with some values falling outside the predicted range. However, this minor discrepancy in T_max_ did not significantly impact the overall drug exposure and was considered acceptable for the predictive use of the model. Despite the minor inconsistency, the overall visual fit between the predicted and observed profiles was good, supporting the validity of the model for DDI predictions.

### 3.2 Population model development

#### 3.2.1 Japanese post-renal transplant population

We created a virtual Japanese post-renal transplant population by modifying the pre-existing healthy Japanese adult population in Simcyp. We used the published data for early renal transplant patients (Satoh et al., 2008) and adjusted the following physiological parameters: serum creatinine (1.27 mg/dL; CV: 34.09%), serum albumin (4.1 g/dL; CV: 25.87%), and hematocrit (30.1%; CV: 14.38%).

Next, we adjusted for the abundance of CYP3A enzymes (Itohara et al., 2022). The mean hepatic and intestinal CYP3A5 abundance were reduced from 82.3 to 20.5 pmol/mg protein and from 20.5 to 7.97 pmol/whole gut, respectively, to accurately reflect the actual physiological state of Japanese patients during the early post-renal transplantation period.

### 3.3 DDI simulations

#### 3.3.1 IR vs. ER-TAC with LET

We compared the DDI effects of LET on IR-TAC and ER-TAC using an established PBPK modeling approach, as previously described (Wang et al., 2019). The simulation conditions are presented in Table 1. AUC_inf_ ratio (with LET/alone) was 3.26 (95% confidence interval: 2.95–3.61) for IR-TAC and 2.72 (95% confidence interval: 2.48–2.99) for ER-TAC, resulting in an ER/IR ratio of 0.83 (Table 5). This result suggests slightly weaker DDI effects of LET on ER-TAC than on IR-TAC. No significant effect of CYP3A5 genotype was observed on the DDI between IR-TAC and ER-TAC.

**Table 5:** Comparison of pharmacokinetic parameters for IR vs. ER-TAC with LET.

	CYP3A5*1			CYP3A5*3/*3			No stratified		
	IR-TAC + LET/ IR-TAC alone	ER-TAC + LET/ ER-TAC alone	ER/IR ratio	IR-TAC + LET/ IR-TAC alone	ER-TAC + LET/ ER-TAC alone	ER/IR ratio	IR-TAC + LET/ IR-TAC alone	ER-TAC + LET/ ER-TAC alone	ER/IR ratio
AUC_inf_ ratio	2.29 (2.01–2.62)	1.90 (1.69–2.13)	0.83	3.51 (3.13–3.94)	2.78 (2.64–3.26)	0.79	3.26 (2.95–3.61)	2.72 (2.48–2.99)	0.83
C_max_ ratio	1.45 (1.33–1.57)	1.45 (1.33–1.59)	1.00	1.62 (1.54–1.71)	1.64 (1.55–1.69)	1.01	1.59 (1.52–1.67)	1.59 (1.53–1.56)	1.00

IR-TAC, immediate-release tacrolimus; ER-TAC, extended-release tacrolimus; LET, letermovir; AUC_inf_, area under the blood concentration–time curve from 0 to infinity; C_max_, maximum concentration.

AUC_inf_ and C_max_ values are represented as geometric means with 95% confidence intervals (95% CIs).

Further analysis of CL values (Table 6) revealed similar inhibition patterns for both formulations in the liver, with a CYP3A4-mediated CL reduction of approximately 53% for both IR-TAC and ER-TAC co-administered with LET. In the small intestine, inhibitory effect on CYP3A4-mediated CL was slightly more pronounced for IR-TAC (47% reduction) than for ER-TAC (48% reduction). Notably, LET had minimal impact on CYP3A5- and UGT1A1-mediated CL of both formulations in all examined tissues.

**Table 6:** Hepatic, small intestine, and colon intrinsic clearance of TAC with or without letermovir (LET) via different pathways.

CLint value (minimum value)	CYP3A4		CYP3A5		UGT1A1	
	IR-TAC	ER-TAC	IR-TAC	ER-TAC	IR-TAC	ER-TAC
Hepatic CLint of TAC (L/h)	8890.4	8892.1	760.5	760.6	35.5	35.4
Hepatic CLint of TAC with LET (L/h)	4155.7	4137.1	760.3	760.5	35.5	35.4
Co-administration/alone ratio	0.47	0.47	1.00	1.00	1.00	1.00
SI CLint of TAC (L/h)	35.2	61.6	1.9	2.7	0.8	0.8
SI CLint of TAC with LET (L/h)	18.6	31.9	1.5	2.6	0.8	0.8
Co-administration/alone ratio	0.53	0.52	0.81	0.96	1.00	1.00
Colon CLint of TAC (L/h)	1.8	2.6	0.1	0.1	0.0	0.0
Colon CLint TAC with LET (L/h)	2.3	2.5	0.1	0.1	0.0	0.0
Co-administration/alone ratio	1.31	0.94	0.83	0.99	N.D.	N.D.

SI, small intestine; TAC, tacrolimus; LET, letermovir; CLint, intrinsic clearance.

#### 3.3.2 ER-TAC with LET in the virtual Japanese post-renal transplant population

In the virtual Japanese post-renal transplant population, co-administration of LET and ER-TAC significantly changed TAC pharmacokinetics, with the effects varying by CYP3A5 genotype and sex (Table 7). For CYP3A5*1 carrier, the AUC_inf_ ratio (ER-TAC + LET/ER-TAC alone) was 2.33 for males and 2.53 for females, indicating a substantial increase in overall TAC exposure. In CYP3A5*3/*3 carriers, this effect was even more significant, with ratios of 2.86 for males and 2.82 for females. Concurrently, C_max_ ratio ranged from 1.49 to 1.51, suggesting a moderate increase in peak TAC concentration. CL ratio decreased to 0.43 and 0.40 for male and female CYP3A5*1 carrier, and 0.35 for both male and female CYP3A5*3/*3 carriers, indicating a marked reduction in TAC elimination.

**Table 7:** Comparison of pharmacokinetic parameters for ER-TAC with and without LET in Japanese early renal transplant patients, stratified by CYP3A5 genotype and sex.

	CYP3A5*1						CYP3A5*3/*3					
	ER-TAC + LET		ER-TAC alone		ER-TAC + LET/ER-TAC alone ratio		ER-TAC + LET		ER-TAC alone		ER-TAC + LET/ER-TAC alone ratio	
	Male	Female	Male	Female	Male	Female	Male	Female	Male	Female	Male	Female
AUC_inf_ (ng/mL*h)	463.43	556.59	198.72	219.74	2.33 (2.09–2.6)	2.53 (2.25–2.85)	684.39	787.05	239.27	278.69	2.86 (2.53–3.24)	2.82 (2.46–3.25)
C_max_ (ng/mL)	9.22	9.13	6.17	6.07	1.49 (1.41–1.59)	1.50 (1.42–1.59)	10.43	9.62	6.92	6.42	1.51 (1.43–1.58)	1.50 (1.41–1.6)
t_max_ (h)	4.00	4.11	4.23	4.48	0.94 (0.75–1.19)	0.94 (0.52–1.12)	3.92	4.05	4.24	4.39	0.95 (0.68–1.16)	0.94 (0.53–1.96)
CL (L/h)	10.79	8.98	25.16	22.75	0.43 (60.00)	0.4 (65.96)	7.31	6.35	20.90	17.94	0.35 (48.64)	0.35 (68.25)

IR-TAC, immediate-release tacrolimus; ER-TAC, extended-release tacrolimus; LET, letermovir; AUCinf, area under the blood concentration–time curve from 0 to infinity; Cmax, maximum concentration. T_max_: time to reach maximum concentration.

AUC_inf_ and C_max_ values are represented as geometric means with 95% confidence intervals (95% CIs). T_max_ values are median (range). CL values are geometric means (% geometric coefficient of variation).

## 4 Discussion

Using a novel PBPK model for ER-TAC, this study revealed two key findings related to the DDIs between LET and TAC in Japanese renal transplant patients.

The first key finding was that the PBPK model accurately predicted the pharmacokinetics of both IR and ER-TAC formulations when co-administered with LET, representing the first report of ER-TAC modeling using Simcyp. Our model development is particularly significant considering the increasing recommendations for the use of ER-TAC in renal transplantation (Kuypers et al., 2013). Our model exhibited excellent predictive performance for ER-TAC, with AUC_0-24_ and C_max_ prediction ratios of 1.12 and 1.14, respectively (Table 4), highlighting its reliability.

The second key finding was that the co-administration of LET with ER-TAC significantly increased the TAC exposure in the virtual Japanese post-renal transplant population, with the effect varying by CYP3A5 genotype, while the impact of sex was minimal (Table 7). For CYP3A5*1 carrier, the AUC_inf_ ratio (ER-TAC + LET/ER-TAC alone) ranged from 2.33 to 2.53, while for CYP3A5*3/*3 carriers, it ranged from 2.82 to 2.86. Concurrently, C_max_ ratios ranged from 1.49 to 1.51 across all groups. The European Society for Organ Transplantation guidelines on the therapeutic drug monitoring of TAC emphasize AUC as a key indicator of TAC efficacy in transplant patients (Brunet et al., 2019). The observed AUC_inf_ ratio indicated that the ER-TAC dose should be reduced by approximately 57–60% (1–1/2.33 = 0.57 to 1–1/2.53 = 0.60) CYP3A5*1 carrier, and 65% (1–1/2.82 = 0.65 to 1–1/2.86 = 0.65) for CYP3A5*3/*3 carriers when co-administered with LET to maintain therapeutic efficacy. In practical terms, this indicates multiplying the standard ER-TAC dose by a factor of 0.40–0.43 for CYP3A5*1 carrier and 0.35 for CYP3A5*3/*3 carriers in the presence of LET. The magnitude of this dose adjustment indicates the need for careful therapeutic drug monitoring and individualized dosing regimens when using medication combinations for Japanese renal transplant patients. Although our model provides general guidance, it should always be used in conjunction with patient-specific factors and clinical responses to make effective dosing decisions. Furthermore, the significant interactions between ER-TAC and LET indicate the need for personalized immunosuppressive therapy approaches for Japanese renal transplant patients.

Notably, interactions of LET with ER-TAC differed from those of LET with IR-TAC. DDI simulations indicated a smaller increase in ER-TAC exposure compared to IR-TAC exposure when co-administered with LET (Table 5). Even when the same dose of TAC is administered, differences in AUC between ER and IR formulations have been observed, likely due to variations in the rate and extent of TAC release in the small intestine (van Gelder et al., 2024). ER-TAC, designed to release the drug more slowly, may result in lower peak concentrations, thereby reducing the degree of CYP3A4 inhibition by LET in the small intestine (van Gelder et al., 2020). On the other hand, this discrepancy cannot be fully explained by the differences in CYP3A5 genotypes and the CYP inhibition rates or abundances (Tables 5, 6). Absence of P-glycoprotein (P-gp) data in our model may have contributed to this unexpected result, suggesting the presence of unknown factors influencing drug interactions, similar to previous reports (Saeki et al., 1993; Hebert, 1997). Our findings highlight the complexity of DDIs, warranting further investigation of the mechanisms underlying formulation-specific interactions.

This study’s results are consistent with those of previous reports on the inhibitory effects of LET on CYP3A4 enzyme (Menzel et al., 2023). However, the magnitude of drug interaction in this study exceeded the typical threshold for clinically significant DDIs (Guest et al., 2011). This finding is especially relevant for Japanese patients owing to the higher frequency of CYP3A5-expressing individuals (CYP3A5*1 allele carrier) in this population than in Caucasians (Satoh et al., 2008). Approximately 50% of Japanese individuals express CYP3A5, which typically results in low TAC exposure due to increased metabolism (Barry and Levine, 2010). Our results, as shown in Table 7, suggest that the effect of LET co-administration on TAC exposure may differ based on CYP3A5 genotype. Although not statistically significant, we observed a trend towards a greater increase in AUC for CYP3A5*3/*3 carriers compared to CYP3A5*1 carrier when LET was co-administered. This observation aligns with the understanding that CYP3A5*3/*3 individuals rely more heavily on CYP3A4 for TAC metabolism, potentially leading to a more pronounced effect of LET’s CYP3A4 inhibition. These findings underscore the importance of our model to predict DDIs in Japanese renal transplant patients. TAC is known to inhibit CYP3A4 and UGT1A1 enzymes, which could potentially affect LET’s pharmacokinetics. Analysis of CL values indicated that the effects of ER-TAC on CYP3A4 and UGT1A1 enzymes did not differ significantly from those of IR-TAC (Table 6). This finding, combined with information from the Japanese LET package insert indicating a minimal impact of IR-TAC on LET pharmacokinetics, suggests that the interaction between ER-TAC and LET is primarily unidirectional. The dose of LET should not be reduced to prevent LET-resistant CMV (Alain et al., 2020). Decreased enzyme inhibition by ER-TAC is possibly due to its ER formulation, which results in low peak concentrations and weak enzyme inhibition. However, these findings need to be validated in future clinical studies to fully elucidate the complex pharmacokinetic interactions between different TAC and LET formulations in diverse patient populations. Here, our model accounted for the effects of hematocrit values on TAC pharmacokinetics, being particularly beneficial for renal transplant patients. Previous studies have suggested that low hematocrit values, often observed in transplant patients, lead to increased TAC CL (Undre and Schäfer, 1998). This physiological change can contribute to differences in TAC pharmacokinetics between healthy individuals and renal transplant patients, further emphasizing the importance of population-specific modeling to accurately predict DDIs.

Our study demonstrates that co-administration of TAC with LET significantly increases TAC exposure, with AUC_inf_ ratios ranging from 2.33 to 2.86 and C_max_ ratios from 1.49 to 1.51 for different CYP3A5 genotypes (Table 7). This increase in ER-TAC exposure raises important clinical considerations. TAC is characterized by a narrow therapeutic index, necessitating careful therapeutic drug monitoring (TDM) to maintain efficacy while minimizing toxicity (Brunet et al., 2019). In renal transplant recipients, achieving and maintaining optimal TAC exposure is particularly challenging due to the dynamic nature of the post-transplant period (Nguyen et al., 2023). The increased TAC exposure observed with LET co-administration may elevate the risk of TAC-related adverse effects, including nephrotoxicity, neurotoxicity, and metabolic disturbances (Xie et al., 2022). Moreover, the required TAC dose to maintain therapeutic levels varies significantly based on the time post-transplantation, with higher doses typically needed in the early post-transplant period and lower doses during maintenance therapy (Satoh et al., 2008). This variability, coupled with the increased exposure due to LET, underscores the need for vigilant monitoring and dose adjustment of TAC when used concomitantly with LET in renal transplant recipients.

It is important to note that our study focused on the oral formulation of LET at its approved fixed dose of 480 mg daily (240 mg when co-administered with cyclosporine). This fixed-dose regimen is the standard clinical practice, designed to minimize the risk of resistance development. However, as highlighted by Wang et al. (2019), LET’s effects on TAC pharmacokinetics may be dose-dependent, and blood exposure could vary significantly between oral and injectable formulations. Our study did not investigate the time- and concentration-dependent CYP3A inhibitory effects of LET, which represents a limitation of our current work. While the fixed-dose regimen simplifies clinical use, it also limits our understanding of dose-related effects. Further research is needed to elucidate these aspects, particularly on the assumption that different dosage forms and dose adjustments. Such investigations could provide valuable insights for optimizing LET use in various clinical scenarios, including both oral and intravenous administration in transplant recipients, while maintaining the benefits of a standardized dosing approach.

This study has some limitations. The developed model was based on *in vitro* data and published literature. Although parts of it was validated against available clinical data, the component for Japanese early renal transplant patients was developed using a bottom-up approach. Prospective clinical trials are necessary to confirm its predictions. Additionally, the model does not account for all potential variability factors, such as comorbidities or concomitant medications, that may be observed in real-world transplant populations. Another notable limitation is the discrepancy in T_max_ prediction with our model, possibly due to the absence of P-gp data. Therefore, future studies incorporating P-gp kinetics are necessary to improve the accuracy of the model in predicting drug absorption profiles. However, the discrepancy in T_max_ values did not significantly affect the overall drug exposure, as both AUC and C_max_ predictions were accurate. Moreover, although hematocrit values were included in our population model, the effect of hematocrit changes on TAC pharmacokinetics could not be comprehensively evaluated, warranting further research and model refinement. Nevertheless, the established model provides a framework to predict DDIs in Japanese renal transplant patients, serving as a valuable tool to optimize immunosuppressive therapy for these patients.

## 5 Conclusion

In this study, PBPK modeling was performed to investigate the DDIs between LET and TAC in Japanese renal transplant patients, taking into account CYP3A5 genotypes. We successfully developed and validated a novel PBPK model for ER-TAC that accurately predicted its pharmacokinetics when co-administered with LET. Our simulations revealed a significant increase in ER-TAC exposure when co-administered with LET in a virtual Japanese post-renal transplant population, with the effect varying by CYP3A5 genotype. For CYP3A5*1 carrier, the AUC_inf_ ratio ranged from 2.33 to 2.53, while for CYP3A5*3/*3 carriers, it ranged from 2.82 to 2.86. C_max_ ratio was approximately 1.50 across all groups. Therefore, when LET is co-administered with ER-TAC for Japanese renal transplant patients, ER-TAC dose should be reduced by approximately 57–65%, depending on the CYP3A5 genotype, to maintain its therapeutic efficacy. These specific insights, which are currently absent from Japanese package inserts, can aid in managing complex drug interactions and optimizing immunosuppressive therapy for Japanese renal transplant patients. Our findings highlight the importance of careful monitoring, consideration of CYP3A5 genotypes, and individualized dosing strategies when using medication combinations for Japanese renal transplant patients. The observed differences in DDI profiles between the IR-TAC and ER-TAC formulations further underscore the importance of formulation-specific considerations in treatment. Moreover, incorporation of population-specific factors, such as hematocrit values, in the developed model facilitates the accurate prediction of drug interactions in renal transplant patients. Overall, this study expands the application of PBPK modeling in transplant pharmacology and suggests methods to optimize immunosuppressive therapy for renal transplant patients.

## Data Availability

The original contributions presented in the study are included in the article/supplementary material, further inquiries can be directed to the corresponding author.
